# The Evolving Landscape of Immunotherapy-Based Combinations for Frontline Treatment of Advanced Renal Cell Carcinoma

**DOI:** 10.3389/fimmu.2018.03120

**Published:** 2019-01-10

**Authors:** Asim Amin, Hans Hammers

**Affiliations:** ^1^Levine Cancer Institute, Charlotte, NC, United States; ^2^Atrium Healthcare System, Charlotte, NC, United States; ^3^University of Texas Southwestern Medical Center, Dallas, TX, United States

**Keywords:** combination immunotherapy, VEGF inhibition, immune check point inhibitor, advanced renal cell carcinoma, immuno modulation

## Abstract

Insights into the biology of advanced renal cell carcinoma (aRCC) and the development of agents targeting the vascular endothelial growth factor (VEGF) pathway have positively impacted the outcomes for patients with aRCC. With the recent approval of the dual immune checkpoint inhibitors (ICIs), nivolumab and ipilimumab, by the U.S. Food and Drug Administration (USFDA), and the European Medicines Agency (EMA), the era of VEGF monotherapy for untreated aRCC appears to be coming to an end for patients with access to the combination therapy. The frontline treatment options for renal cell carcinoma are evolving rapidly and will lead to the approval of other combination immunotherapies—especially those with VEGF inhibitors. Here we review the clinical data for dual immune checkpoint inhibition with nivolumab plus ipilimumab as well as the emerging data for ICI plus VEGF inhibitor combinations and discuss the challenges these will pose for the clinical practitioner.

## Introduction

### Historical Perspective

Immunotherapy with high-dose interleukin-2 (HD IL-2) (1, 2) had been the mainstay for treatment of advanced renal cell carcinoma (aRCC) in the United States until agents targeting the VEGF pathway became available in 2005. HD IL-2 was shown to elicit a response in 25% of patients with advanced clear renal cell carcinoma (3). **PR**oleukin **O**bservational Study to Evaluate the Treatment Patterns and **CL**inic**a**l Response in **M**alignancy (PROCLAIM), a US-based multicenter study designed to capture real-world clinical data for interleukin-2 in patients with metastatic melanoma, aRCC, or other malignancies showed that response after treatment with HD IL-2 was durable. The median overall survival was not reached at a median follow-up of 21 months; the 30-month survival rate for patients who achieved a complete response (CR), partial response (PR), or had stable disease (SD) was 100, 75, and 78%, respectively (4). Given the considerable toxicity associated with HD IL-2, the applicability and overall impact on kidney cancer was limited since it required patients to have an overall excellent level of fitness and specially trained staff to oversee administration. The advent of multi-tyrosine kinase inhibitors (TKIs) or antibodies targeting the VEGF axis clearly had a significant and broad impact on the natural history of advanced renal cell carcinoma; however, durable response is rare (5–14), and therefore the development of new options that are tolerable and have the potential for durable responses remains an area of active investigation.

### Anti-tumor Immune Response

**Figure 1:**
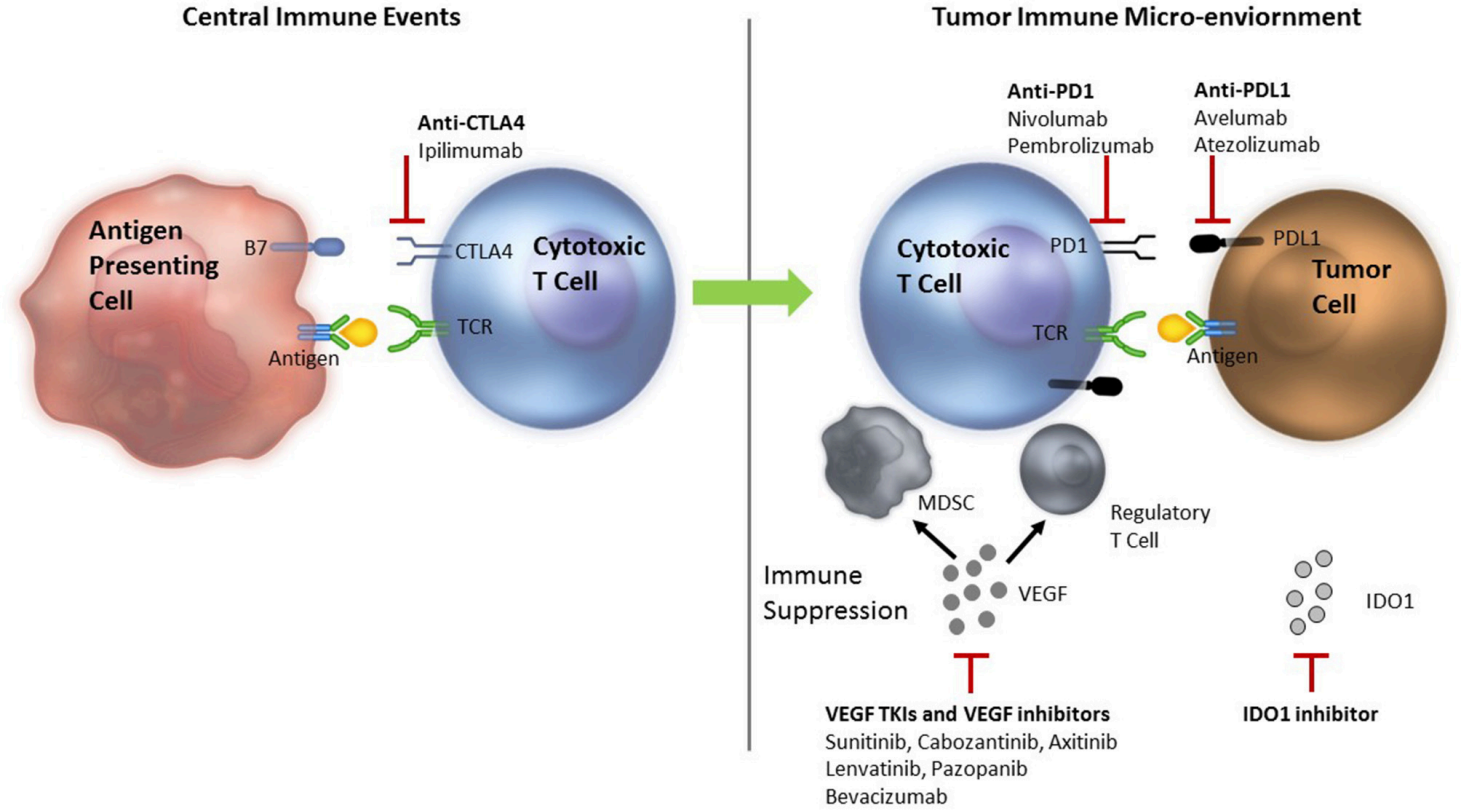
Rationale for combining ICIs with VEGF inhibitors—Anti-tumor immune response modulation. TCR, T cell receptor; MDSC, myeloid derived suppressor cells; Treg, T regulatory; VEGF, vascular endothelial growth factor; PD-1, programmed death 1; PD-L1, programmed death ligand 1; CTLA 4, cytotoxic T lymphocyte antigen 4; TIM3, T cell immunoglobulin mucin receptor 3; LAG3, lymphocyte activation gene 3; ICIs, immune checkpoint inhibitors. Tumor antigen is presented to the cytotoxic T cells in the lymphoid tissues to initiate an anti-tumor immune response. The response is modified by several ICIs. VEGF inhibits dendritic cell maturation decreasing antigen presentation and inhibits T cells leading to their exhaustion. Primed T cells move to the tumor microenvironment where they encounter more immune suppression induced by VEGF that recruits MDSCs and Treg cells. VEGF also results in neo-angiogenesis that can alter the quality and quantity of infiltrate of the tumor immune microenvironment adding to immune suppression. Inhibition of VEGF by VEGF TKIs and anti-VEGF antibodies can reverse the VEGF induced immune suppression. Anti-CTLA-4 antibodies, ipilimumab and tremelimumab bind to the CTLA-4 inhibitory checkpoint and prevent the T cells from switching off. Anti-PD-1 antibodies nivolumab or pembrolizumab and anti-PD-L1 antibodies avelumab, atezolizumab or durvalumab bind to PD-1 and PD-L1 in the tumor, respectively to prevent T cells from switching off.

The generation of an effective anti-tumor immune response requires several critical events to happen in a well-orchestrated sequence. The initial step is presentation of tumor antigen/s by the dendritic cells/antigen presenting cells in the context of self major histocompatibility complex (MHC) molecules to the T cells. This occurs in the lymphoid tissues or the central immune environment. Recognition of the tumor antigens as non-self by the T cells results in generation of the first signal for an anti-tumor immune response to proceed. T cell activity is subsequently modulated by several proteins—immune checkpoints—expressed on the surface of T cells. These immune checkpoints can serve as both, “on” or “off switches” for the T cell. Blockade of the “off switches” or stimulation of the “on switches” can result in increased activity of the T cells and has been used for modulation of the anti-tumor immune response in the clinical setting (Figure 1).

Cytotoxic T lymphocyte antigen 4 (CTLA-4) is an inhibitory immune checkpoint expressed on the surface of activated T cells. Engagement of CTLA-4 with B-7 family of molecules expressed on the antigen-presenting cells results in an inhibitory signal that switches T cells off. Ipilimumab is an anti-CTLA-4 antibody that blocks this interaction and prevents T cells from switching off (Figure 1) in the central immune environment. Tremelimumab is another anti-CTLA-4 antibody that is currently undergoing investigation. Additionally, T regulatory (Treg) cells, which are potent suppressors of the immune response, are known to express very high levels of CTLA-4 and are probably affected by CTLA-4 inhibition as well. The activated T cells then move to the tumor micro-environment where they encounter multiple inhibitory factors. Programmed death 1 (PD-1) is an inhibitory checkpoint expressed on activated T cells that when engaged with its ligands, PD-L1 or PD-L2 (expressed on some tumors, immune system cells, and normal cells), results in suppression of T cell activity. Blockade of PD-1/PD-L1 axis by anti-PD-1 antibodies (pembrolizumab and nivolumab) or anti-PD-L1 antibodies (atezolizumab, avelumab, and durvalumab) allow T cells to maintain their anti-tumor activity in the tumor micro-environment. Objective responses ranging from 20 to 60% have been demonstrated with ICIs in various tumors.

### ICIs in aRCC

Nivolumab is an anti-PD-1 antibody that blocks the interaction between PD-1 and its ligands PD-L1/PD-L2, thereby preventing the cytotoxic T cells from “switching off” or getting “exhausted.” In a randomized phase 2 study for patients who had previously shown progression after at least one line of anti-angiogenic therapy for their aRCC, nivolumab monotherapy at the doses of 0.3, 2, and 10 mg/kg administered intravenously every 2 weeks, showed an overall response of 20% (15). Treatment was tolerated well and the overall survival compared very favorably to what had been observed in prior phase 3 studies. A subsequent phase 3 (CheckMate 025) study compared nivolumab monotherapy to everolimus in patients with aRCC who had received prior treatment with a VEGF TKI. Statistically significant improvement in overall survival in favor of nivolumab (25 months, 95% CI 21.8-not estimable vs. 19.6 months with everolimus, 95% CI 17.6–23.1) led to the approval of nivolumab for this population of patients with aRCC (hazard ratio 0.73, 98.5% CI 0.57–0.93; *p* = 0.002). Grade 3–4 treatment-related adverse events were observed in 19% of the patients who received nivolumab and 37% who received everolimus (16).

Ipilimumab is an anti-CTLA-4 antibody that prevents activated cytotoxic T cells from “switching off” by blocking its interaction with the B7 family of molecules. In advanced melanoma, the combination of nivolumab plus ipilimumab showed substantial activity and was approved by the USFDA (17, 18). The recent approval of nivolumab plus ipilimumab for intermediate and poor-risk patients with aRCC based on the CheckMate 214 study (described below) in the first-line setting by the USFDA and EMA marked a new milestone and established the proof of concept for combination immunotherapy in aRCC (19), albeit with a modified dosing schema.

## CheckMate 214—Nivolumab Plus Ipilimumab vs. Sunitinib

In this phase 3 study, patients with aRCC were randomized in a 1:1 ratio to receive either nivolumab 3 mg/kg plus ipilimumab 1 mg/kg (N3I1) intravenously for 4 doses every 3 weeks followed by nivolumab monotherapy maintenance every 2 weeks or sunitinib at the dose of 50 mg orally once a day on a 4-week-on, 2-week-off schedule. The co-primary endpoints were objective response rate (ORR), progression free survival (PFS), and overall survival (OS) in intermediate and poor-risk patients. Secondary endpoints were ORR, PFS, and OS in the intention to treat (ITT) population and the incidence of adverse events (20).

One thousand ninety-six patients were enrolled in the study, 550 on the N3I1 arm and 546 on the sunitinib arm; 425 patients in the N3I1 arm and 422 patients in the sunitinib arm were intermediate and poor risk. In the intermediate and poor-risk patients, the ORR was 42% (95% CI 37–47) in the N3I1 arm, compared to 27% (95% CI 22–31) for those who received sunitinib (*p* < 0.001). Statistically significant improvement in overall survival was noted in favor of the N3I1 arm, compared to sunitinib (hazard ratio, 0.63; *p* < 0.001). At a median follow-up of 25.2 months, the median overall survival was not reached for the N3I1 arm (95% CI 28.2 months to not estimable), compared to 26 months for the sunitinib arm (95% CI 22.1 months to not estimable). The median duration of response in the N3I1 arm was not reached (21.8 months to not estimable) and was 18.2 months (14.8 months to not estimable) in the sunitinib arm. The median PFS was 11.6 months, compared to 8.4 months for N3I1 and sunitinib arms, respectively, and did not meet criteria for statistical significance (hazard ratio, 0.82; *p* = 0.03).

No new safety signals were noted; 93% of the patients who received N3I1 and 97%, who were treated with sunitinib, experienced an adverse event. Grade 3–4 events were observed in 46% of the patients in the N3I1 arm and 63% in the sunitinib arm. Treatment was discontinued in 22% of the patients in the N3I1 arm and 12% in the sunitinib arm, secondary to adverse events. There were 8 deaths in the N3I1 arm and 4 deaths in the sunitinib arm attributed to treatment. Around 35% of patients required treatment with high-dose corticosteroids (defined as 40 mg prednisone equivalents for at least 14 days). Based on the above results, the combination of nivolumab and ipilimumab was approved for the first-line treatment of intermediate and high-risk patients with aRCC by the USFDA and EMA.

## Rationale for Combining ICIs With VEGF Inhibition

### VEGF and Tumor Immune Micro-Environment (TIME)

Tumor micro-environment is complex and not well-characterized. Interactions between the milieu of cytokines present in the micro-environment, phenotype of the immune cells, proteins expressed on the tumor cells, stromal components, and vascularity may all impact the outcomes for immunotherapy. VEGF plays a key role in aRCC and has been targeted successfully with significant therapeutic efficacy. The antitumor activity of VEGF TKIs/VEGF blockers has in most part been attributed to inhibition of neo-angiogenesis; however, the angiogenic activity also interacts with the immune status. Therefore, VEGF inhibition may modulate the host tumor immune micro-environment (TIME) and contribute to anti-tumor activity.

The presence of VEGF in the tumor micro-environment can lead to immune suppression via several mecahnisms. High VEGF levels lead to an abnormal vasculature in the tumors with high interstitial pressures that can decrease the immune cell traffic impacting the quantity and quality of the infiltrate. Based on the early data from evaluation of the immune infitrate in the tumor, the TIME has been classified as Binnewies et al. (21):

I-E TIME (Infitrated-excluded): Tumor-immune micro-environment characterized by exclusion of cytotoxic T cells from the core. Considered immunologically “cold tumors.”
  I-I TIME (Infitrated-inflamed): Tumor-immune micro-environment infiltrated with cytotoxic T lymphocytes expressing PD-1, leukocytes and tumor cells expressing PD-L1. Considered immunologically “hot tumors.”
  TLS-TIME (Infiltrated-inflamed tertiary lymphoid structures): A subclass of infiltrated–inflamed micro-environment displaying tertiary lymphoid structures/aggregates with every population of lymphocytes including naïve T cells, regulatory T cells, B cells, and dendritic cells.

The quality and quantity of immune cell infiltrate in the TIME can impact the response to immunotherapy with ICIs. Immunologically “hot” tumors respond more often than“cold” tumors.

Increased level of VEGF in the tumor can induce suppression of both innate and adaptive immune responses, e.g., VEGF has been shown to directly inhibit dendritic cell maturation (22, 23), increase recruitment of myeloid-derived suppressor cells (MDSCs) and Treg cells (24, 25), and decrease trafficking and efficacy of cytotoxic T cells (26). VEGF has also been reported to inhibit T cell development (27). VEGF-A in the tumor micro-environment was shown to increase expression of inhibitory checkpoints, PD-1, CTLA-4, TIM3, and LAG3, which was shown to be reversed by antibodies against VEGFR-2 (26). Elevated serum and tumor VEGF levels have been associated with poor disease-specific survival in patients with aRCC (28).

Inhibition of the VEGF axis by VEGF TKIs and anti-VEGF antibodies can potentially reverse the immune suppression induced by VEGF. In the preclinical renal cell carcinoma model (RENCA), combination of a murine anti-PD-1 antibody and suntinib showed synergistic activity and greater numbers of tumor infiltrating lymphocytes, compared with controls treated with each agent alone (29). In a clinical trial, patients with aRCC showed significant increase in the percentage of interferon gamma–producing T cells, decrease in IL-4 production, and decrease in Treg cells in the peripheral blood after receiving sunitinib 50 mg orally once a day for 28 days (30). Significant reduction in MDSCs was also observed, demonstrating reversal in immune suppression (31). Expansion of tumor infiltrating lymphocytes and reduction in MDSCs was observed in primary tumors from patients who received sunitinib prior to the surgery, compared to those who were treatment-naïve (32). Insight into how different TKIs may vary in their ability to modulate the TIME is still limited. While sunitinib did not appear to impact dendritic cell function, sorafenib was noted to inhibit generation of antigen-specific T cells due to dendritic cell suppression (33). Bevacizumab has been noted to promote activity and reverse the inhibitory effects of VEGF on dendritic cells (34, 35). In a clinical trial with the combination of ipilimumab plus bevacizumab in patients with advanced melanoma, activated vessel endothelium with extensive CD8+ cell and macrophage infiltration was observed, compared to controls who received ipilimumab alone (36).

Durable responses have been observed with immune checkpoint inhibitors (ICIs) in up to 40% of the patients with aRCC treated with a combination of PD-1/CTLA-4 inhibitors. Although there has been a significant improvement compared to historical controls, there remains an unmet need in this therapeutic area. Recognizing that there are big gaps in our knowledge, VEGF inhibitors by improving dendritic cell function, antigen presentation, normalization of the tumor vasculature with greater trafficking of immune cells, increased cytotoxic T cell infiltration, and decreased MDSCs and Treg cells could potentially reduce the immunosupressive effect in the tumor micro-environment; therefore, evaluating them in combination with ICIs appears to be a logical step. In this context, multiple efforts are underway; here we describe the immune checkpoint inhibitor–based combinations that are approved or are in advanced stages of development (Tables 1, 2).

**Table 1:** Phase 1/2 immunotherapy based combination studies.

**Study**	**Patients**	**ORR**	**PFS**
CheckMate 016 (Phase 1)		
N+S	*n* = 33	54.5%	12.7 mon (95% CI, 11.01–16.66)
N+P	*n* = 20	45%	7.2 mon (95% CI, 2.79–11.07)
N1I3	*n* = 47	40.4%	9.4 mon (95% CI, 5.6–18.6)
N3I1	*n* = 47	40.4%	7.7 mon (95% CI, 3.7–14.3)
NCT01472081		
Pembrolizumab + Axitinib	*n* = 52	73%	20.9 mon (95% CI, 15.4–not evaluable)
(Phase 1/2)		(8% CR)
NCT02133742		
IMmotion 150 (RP2)	*n* = 305		
Atezolizumab + Bevacizumab	*n* = 101	Atezolizumab + Bevacizumab
vs. Sunitinib vs. Atezolizumab		ITT = 32%	11.7 mon (95% CI, 8.4–17.3)
		PD-L1+ = 46%	14.7 mon (95% CI, 8.5–25.1)
	*n* = 100	Sunitinib ITT = 29%	8.4 mon (95% CI, 7.0–14)
		PD-L1+ = 27%	7.8 mon (95% CI, 3.8–10.8)
	*n* = 103	Atezolizumab ITT = 25%	6.1 mon (95% CI, 5.4–13.6)
NCT01984242		PD-L1+ = 28%	5.5 mon (95% CI, 3.0–13.9)
JAVELIN Renal 100 (Phase 1b)	*n* = 55	ITT = 58.2% (5.5% CR)	Not available
First Line Avelumab + Axitinib		PD-L1≥1% vs. PD-L1- 65.9% and 36.4%
NCT02493751		PD-L1≥5% vs. PD-L1- 67.9% and 50%
Study 111 Pembrolizumab + Lenvatinib	*n* = 30	66.7%	17.7 mon (95% CI, 9.6–not estimable)
(Phase 1b/2) NCT 02501096	Treatment naïve *n* = 12	83%
	Previously treated *n* = 18	50%
	PD-L1 +	58%
	PD-L1-	71%
Nivolumab + Cabozantinib	*n* = 7		Not available
		54%
Nivolumab + Cabozantinib + Ipilimumab (Phase1)	*n* = 6	

*ORR, Objective response rate; PFS, Progression free survival; N+S, Nivolumab plus Sunitinib; N+P, Nivolumab plus Pazopanib; N1I3, Nivolumab 1 mg/kg plus Ipilimumab 3 mg/kg; N3I1, Nivolumab 3 mg/kg plus Ipilimumab 1 mg/kg; RP2, Randomized Phase 2*.

**Table 2:** Phase 3 immunotherapy based combination studies.

**Study**	**Patients**	**ORR**	**PFS**
CheckMate 214 (Phase 3)	Total *n* = 1096		
First line		
N3I1 → N vs. Sunitinib	Intermediate and poor risk	42% (9% CR)	11.6 mon (95% CI, 8.7–15.5)
	*n* = 425		Hazard ratio – 0.82 (99.1% CI, 0.64–1.05) *p* = 0.03
	Intermediate and poor risk *n* = 422	27% (1% CR)	8.4 mon (95% CI, 7.0–10.8)
N3I1 → N vs. Sunitinibn	ITT *n* = 550	39%	12.4 mon (95% CI,9.9–16.5)
			Hazard ratio – 0.98 (99.1% CI, 0.79–1.23) *p* = 0.85
			12.3 mon (95% CI, 9.8–15.2)
	ITT *n* = 546	32%
NCT02231749			
IMmotion 151	ITT = 915		
(Phase 3)	PD-L1+ *n* = 362		
First Line			
Atezolizumab + Bevacizumab vs. Sunitinib	ITT *n* = 454	37% (CR 5%)	11.2 mon (95% CI, 9.6–13.3)
	PD–L1+ *n* = 178	43% (CR 9%)	11.2 mon (95% CI, 8.9–15)
	ITT *n* = 461	33% (CR 2%)	8.4 mon (95% CI, 7.5–9.7)
	PD-L1+ *n* = 184	35% (CR 4%)	7.7 mon (95% CI, 6.8–9.7)
			Hazard ratio for PD-L1+ patients – 0.74 (95% CI, 0.57–0.96), *p* = 0.02
NCT02420821			
JAVELIN Renal 101 (Phase 3)	Total *n* = 886		
First Line			
Avelumab + Axitinib vs. Sunitinib	ITT *n* = 442	51.4%	13.8 mon (95% CI, 11.1–not estimable)
	PD-L1+ *n* = 270	55.2%	13.8 mon (95% CI, 11.1–not estimable)
	ITT *n* = 444	25.7%	8.4 mon (95% CI, 6.9–11.1)
	PD-L1+ *n* = 290	25.5%	7.2 mon (95% CI, 5.7–9.7)
			Hazard ratio for PD-L1+ patients – 0.61 (95% CI, 0.47–0.79), *p* < 0.0001
			Hazard ratio for ITT patients – 0.69 (95% CI, 0.56–0.84), *p* = 0.0001
NCT02684006			

*ORR, Objective response rate; PFS, Progression free survival; N3I1 → N, Nivolumab 3 mg/kg plus Ipilimumab 1 mg/kg followed by Nivolumab maintenance*.

## Immunotherapy Plus VEGF Inhibitor Combination Studies

### CheckMate 016—Nivolumab Plus TKIs (Sunitinib/Pazopanib) or Ipilimumab

CheckMate 016 was the first trial to explore the safety and tolerability of combination immunotherapy in the setting of aRCC (37–39). This multicenter phase 1 study had 5 treatment arms that included the combination of nivolumab (N) with either TKIs, sunitinib (S) or pazopanib (P) or ipilimumab (I) at the following doses; nivolumab 1 mg/kg plus ipilimumab 3 mg/kg (N1I3), nivolumab 3 mg/kg plus ipilimumab 1 mg/kg (N3I1), and nivolumab 3 mg/kg plus ipilimumab 3 mg/kg (N3I3). The TKI combination arms had an initial dose escalation phase with a starting nivolumab dose of 2 mg/kg (N2) intravenously every 3 weeks with planned increase to nivolumab 5 mg/kg (N5) intravenously every 3 weeks in the expansion phase dependent on the maximum tolerated dose (MTD) assessed by the modified toxicity probabity interval (MTPI). Sunitinib and pazopanib in these arms were administered at the standard dose of 50 mg orally once a day on a 4-week-on, 2-week-off schedule, and 800 mg orally once a day, respectively. In the nivolumab plus ipilimumab arms, the combination therapy with N1I3, N3I1, or N3I3 was administered intravenously every 3 weeks for 4 doses in the induction phase followed by maintenance nivolumab monotherapy at the dose of 3 mg/kg, administered intravenously every 3 weeks. Primary endpoints for the study were safety and tolerability; secondary endpoints included ORR, duration of response (DOR), and PFS rate.

A total of 194 patients were enrolled in this study, 153 received treatment; 33 patients received N+S; 20 recieved N+P; 47 patients were assigned to both N1I3 and N3I1 arms; and 6 patients were treated on the N3I3. The N+S arm completed dose-escalation and expansion phases, while the N+P arm did not proceed to expansion given the early hepatic toxicity observed in the dose-escalation phase. All 6 patients in the N3I3 arm were censored at the time of analysis.

All patients (100%) assigned to the ICI+VEGF–TKI combination arms, N+S and N+P, experienced a treatment-related adverse event; the incidence of grade 3–4 events was 81.8 and 70%, respectively. The most common grade 3–4 adverse events were hypertension (18.2%, 10%), increased alanine aminotrasferase (ALT) (18.2%, 20%), increased aspartate aminotransferase (AST) (9.1%, 20%), diarrhea (9.15, 20%), and fatigue (9.1%, 15%) in the N+S and N+P arms, respectively. In the N+S arm, 39.4%, and in the N+P arm, 60%, of the patients required systemic corticosteroids for management of adverse events that were attributed to immune-mediated etiology. There were no deaths attributed to treatment; 39.4% of the patients in N+S arm and 25% in the N+P arm discontinued treatment due to adverse events.

In the ICI combination arms, N3I1 and N1I3, 93.6% of patients experienced treatment-related adverse event; 50% of these events were graded as 3–4. The incidence of grade 3–4 events in the N3I1 arm was 38.3% and in the N1I3 arm, 61.7%. The most common grade 3–4 adverse events were diarrhea (4.3, 14.9%), increased AST (4.3, 12.8%), ALT (4.3, 21.3%), and asymptomatic elevation of lipase (14.9, 27.7%), respectively. Any treatment with corticosteroids was required in 61.7% patients in the N3I1 arm and 83% of the patients in the N1I3 for management of immune-mediated adverse events. No deaths were attributed to treatment in either arm; 10.6 and 27.7% of the patients in the N3I1 and N1I3 arms, respectively, discontinued treatment due to adverse events.

The confirmed ORR was 54.5% in the N+S arm and 45% in the N+P arm. Responses were sustained; the median DOR in the N+S arm was 60.2 weeks (37.1–not reached) and 30.1 weeks (12.1–174.1) in the N+P arm. The median PFS was 12.7 months (11–16.7) for the N+S arm and 7.2 months (2.8–11.1) for the N+P arm. At a median follow-up of 50.0 months, the median OS was not reached (36.8–NR) in the N+S arm and was 27.9 months (13.3–47.0) in the N+P arm.

The confirmed ORR was 40.4% in the ICI combination arms, N3I1 and N1I3; 10.6% in the N3I1 arm achieved a complete response. The median PFS was 7.7 months (95% CI 3.7–14.3) in the N3I1 arm and 9.4 months (95% CI 5.6–18.6) in the N1I3 arm. The median OS was not reached in the N3I1 arm (95% CI 26 months to not reached) and was 32.6 months (95% CI 26 months to not reached) in the N1I3 arm.

The N3I1 combination arm was observed overall to have the most favorable toxicity profile and efficacy that led to the phase 3 CheckMate 214 trial, comparing this dose to standard sunitinib.

### Pembrolizumab Plus Axitinib

An open-label, multicenter phase 1b study reported by Atkins and colleagues evaluated the combination of pembrolizumab at the dose of 2 mg/kg, administered intravenously every 3 weeks plus a starting dose of axitinib at 5 mg orally twice a day in treatment-naive patients with advanced renal cell carcinoma. The study was conducted in 2 phases, an initial dose-finding phase followed by an expansion phase. The primary endpoint was assessment of dose limiting toxicity (DLT) in the first 6 weeks. Secondary endpoints included assessment of adverse events, PD-L1 status, and antitumor activity including best overall response rate (BORR), DOR, PFS, and OS (40).

Eleven patients were treated in the dose-finding phase; 41 patients received treatment in the expansion phase. Of the 11 patients treated in the dose-finding phase, 3 DLTs were observed. MTD was determined to be pembrolizumab 2 mg/kg, every 3 weeks plus axitinib 5 mg orally twice daily, and used for the expansion phase. Grade 3–4 treatment-related adverse events were observed in 65% of the patients; the most common were hypertension (23%), diarrhea (10%), fatigue (10%), and increased ALT (8%). The most common possibly immune-related adverse events observed were diarrhea (29%), increased ALT (13%), hypothyroidism (13%), and fatigue (12%); 19% had grade 3 events.

Objective response was observed in 73% of the patients; 8% had CR, 65% had a PR, and 15% had SD. The median PFS was 20.9 months (95%CI 15.4—not evaluable). The median DOR was 18.6 months (95% CI 15.1—not reached). The median OS was not reached at median follow-up of 20.4 months.

The experience from this study led to the phase 3 KEYNOTE 426 study (NCT02853331), which compared the efficacy and safety of the combination of pembrolizumab plus axitinib to standard sunitinib, administered at the dose of 50 mg orally once a day on a 4-week-on, 2-week-off schedule. This phase 3 study has completed accrual, results are not reported yet (41). A recent press release from the sponsor indicated that at the time of first interim analysis, the combination of pembrolizumab and axitinib met the primary endpoints of improved OS and PFS compared to sunitinib (www.mrknewsroom.com, accessed October 18, 2018).

### Atezolizumab Plus Bevacizumab

Atezolizumab is a humanized IgG1 antibody that binds to PD-L1 and blocks its interaction with PD-1, preventing T cell exhaustion. A phase 1 study of atezolizumab with bevacizumab showed the combination to be safe with similar antitumor activity, as observed in historical controls. IMmotion 150 was a randomized phase 2 study that compared the efficacy of atezolizumab alone, atezolizumab plus bevacizumab vs. standard sunitinib. Atezolizumab was administered at a fixed dose of 1,200 mg intravenously every 3 weeks as monotherapy or with bevacizumab at 15 mg/kg intravenously every 3 weeks in the combination arm. Sunitinib was administered at the dose of 50 mg orally once a day on a 4-week-on, 2-week-off cycle. The primary objective was evaluation of PFS between the atezolizumab containing arms vs. the sunitinib arm based on the PD-L1 expression status (<1 or ≥1%) on the tumor-infiltrating immune cells (42).

A total of 305 patients were accrued at multiple sites between January 2014 and March 2015 in a 1:1:1 ratio. The patient demographics were well-balanced in the 3 arms. At a median follow-up of 20.7 months, the median PFS in the ITT population was 11.7 months (95% CI, 8.4–17.3) in the atezolizumab plus bevacizumab arm, vs. 8.4 months (95% CI, 7.0–14.0) with suntinib (hazard ratio 1.00; 95% CI. 0.60–1.45), and 6.1 months (95%CI, 5.4–13.6) with atezolizumab monotherapy (hazard ratio 1.19; 95% CI, 0.82–1.71 vs. sunitinib). In the PD-L1+ patients, the PFS was 14.7 months (95% CI, 8.2–25.1) for the combination, vs. 7.8 months (95% CI, 3.8–10.8) with sunitinib (hazard ratio 0.64; 95% CI, 0.38–1.08), and 5.5 months (95% CI 3.0–13.9) with atezolizumab monotherapy (hazard ratio 1.03; 95% CI 0.63–1.67 vs suntinib). In the ITT population, the ORR was 32% (CR 7%) for the combination, 29% (CR 5%) for sunitinib, and 25% (CR 11%) for the atezolizumab monotherapy. In the PD-L1+ patients, the ORR was 46% (CR 12%) for the combination, 27% (CR 7%) for sunitinib, and 28% (CR 15%) with atezolizumab monotherapy.

Treatment-related grade 3–4 adverse events were observed in 57% of the patients who recieved suntinib, 17% with atezolizumab, and 40% with the combination. There were 2 treatment-related deaths each in the sunitinib and atezolizumab monotherapy arms, and 3 in the combination arm.

The above experience led to the phase 3 study IMmotion 151 (NCT02420821), which compared the combination of atezolizumab plus bevacizumab vs. suntinib. The preliminary data for this study were presented at the American Society of Clinical Oncology (ASCO)—Genitourinary Conference 2018 (43). This study randomized 915 treatment-naive patients with aRCC in a 1:1 fashion to recieve atezolizumab 1,200 mg intravenously, with bevacizumab 15 mg/kg intravenously every 3 weeks or sunitinib 50 mg orally once a day on a 4-week-on, 2-week-off schedule. Patients were stratified by PD-L1 expression (<1 or ≥1%) on the tumor-infiltrating immune cells. Co-primary endpoints were PFS in PD-L1+ patients and OS in ITT patients. Secondary endpoints were PFS in ITT population, ORR, and DOR (20).

The ITT population included 915 patients; of these, 362 were PD-L1+. The median PFS for the PD-L1+ patients at median follow-up of 15 months was 11.2 months (95% CI, 8.9–15) in the combination arm vs. 7.7 months (95% CI, 6.8–9.7) for sunitinib (HR 0.74; 95% CI 0.57, 0.96, *p* = 0.0217). In the ITT population, the median PFS was 11.2 months (95% CI, 9.6–13.3) for the combination vs. 8.4 months (95% CI, 7.5–9.7) in the sunitinib arm (HR 0.83; 95% CI 0.70,0.97, *p* = 0.0219). The ORR was 43% in the PD-L1+ patients who received the combination vs. 35% for sunitinib. In the ITT population, the ORR was 37% for the combination vs. 33% for sunitinib. OS had not matured at the time of the analysis. Grade 3–4 adverse events were observed in 40% of the patients who received the combination, and 54% who received sunitinib; 12% in the combination arm and 8% in the sunitinib arm discontinued treatment secondary to adverse events. This study met the primary endpoint of improved PFS in the PD-L1+ patients treated with the combination of atezolizumab and bevacizumab compared to sunitinib and supports its use in the frontline setting for these patients.

### Avelumab Plus Axitinib

Avelumab is a human IgG1 antibody that binds to PD-L1 on tumor cells and blocks its interaction with PD-1 expressed on T cells, thereby preventing the T cells from being switched off in the tumor micro-environment. Avelumab has been approved for the treatment of merkel cell carcinoma. The JAVELIN—Renal 100 was a phase 1b dose-finding study that assessed the MTD and safety of the combination of avelumab and axitinib in treatment-naive patients with advanced renal cell carcinoma. In the dose-finding phase, patients recieved axitinib 5 mg orally twice a day and were then initiated on avelumab 10 mg/kg intravenously every 2 weeks. Almost all of the patients in the dose expansion cohort were favorable or intermediate risk. The primary endpoint was evaluation of DLT for the combination in the first 4 weeks of treatment. Secondary enpoints included assessment of safety, ORR, DCR, DOR, PFS, and OS (44).

Of the 79 patients screened between October 2015 and September 2016, 55 were deemed eligible; 6 patients were treated in the dose-finding cohort, and 49 in the expansion cohort. The MTD for the combination was established to be avelumab 10 mg/kg intravenously every 2 weeks plus axitinib 5 mg orally twice a day. Ninety-six percent of the patients experienced at least one adverse event attributed to their treatment; 58% had grade 3–4 events. There was one treatment-related death, secondary to autoimmune myocarditis. Immune-mediated adverse events were observed in 42% of patients; 9% had grade 3–4 severity. Objective response was confirmed in 100% of the patients in the dose-finding cohort and in 53% of the patients in the expansion cohort for an ORR of 58%, 6% being complete responses. At a median follow-up of 52.1 weeks, DOR, PFS, and OS could not be assessed. PD-L1 expression was ascertained for 52 patients. Using a 1% cutoff for expression of PD-L1 on the tumor-associated immune cells, the ORR was 63% for those with expression ≥1%, compared to 36% for those with expression <1%.

The phase 3 study (NCT02684006) JAVELIN—Renal 101 compared the combination of avelumab 10 mg/kg intravenously every 2 weeks plus axitinib 5 mg orally twice a day, vs. sunitinib 50 mg orally once a day on a 4-week-on, 2-week-off schedule for patients with treatment-naive aRCC (45). The preliminary results were reported recently at the ESMO annual meeting 2018 (43). The primary endpoints of this study were PFS by blinded independent central review (BICR) and OS in patients with PD-L1+ (≥1 of immune cells). Secondary endpoints included PFS and OS irrespective of PD-L1 expression, objective response (OR), and safety.

A total of 886 patients were randomized; 442 to the combination of avelumab plus axitinib and 444 to the sunitinib arm. Of the 442 patients in the combination arm, 270 were PD-L1+; 290 patients were PD-L1+ in the sunitinib arm. The median PFS in the PD-L1+ tumors was 13.8 months (95% CI, 11.1—not estimable) for the combination vs. 7.2 months (95% CI, 5.7–9.7) in the sunitinib arm (HR = 0.61; 95% CI, 0.475–0.790; *p* < 0.0001). The median PFS irrespective of PD-L1 status was 13.8 months (95% CI, 11.1—not estimable) for the combination vs. 8.4 months (95% CI, 6.9–11.1) in the sunitinib arm (HR = 0.69; 95% CI, 0.563–0.840; *p* = 0.0001). The OS was immature at the time of data cutoff and reporting.

This study met the primary endpoint of improved PFS in treatment-naive patients with PD-L1+ tumors, and supports the combination of avelumab plus axitinib for treatment of patients with aRCC in the first-line setting.

### Pembrolizumab Plus Lenvatinib

Lenvatinib is a multi-tyrosine kinase inhibitor with activity against VEGF receptors VEGFR1, VEGFR2, VEGFR3; fibroblast growth factor receptors FGFR1, FGFR2, FGFR3, FGFR4; and platelet-derived growth factor alpha, KIT, and RET. Based on a randomized phase 2 study the combination of lenvatinib plus everolimus was approved for the treatment of aRCC in the second-line setting. A preclinical study with Lenvatinib showed decrease in the macrophage population within the tumor micro-environment that correlated with increased antitumor activity with PD-1 inhibition (46). In a multicenter, open-label phase 1b study, 8 patients with aRCC that had progressed after standard treatment were treated with the combination of lenvatinib plus pembrolizumab. The initial starting dose of lenvatinib was 24 mg/day that was decreased to 20 mg/day due to toxicity. Pembrolizumab was administered at the dose of 200 mg intravenously every 3 weeks. Phase 2 of this study included 22 patients with aRCC who could have received up to 2 prior lines of treatment. The primary endpoint for the phase 2 cohort was ORR at 24 weeks. Secondary endpoints included progression-free survival and duration of response (47, 48).

Of the 30 patients included in the phase 1b/2 study, PD-L1 status was assessed for 26 patients; 12 were PD-L1-positive using 1% cutoff. Eighteen patients had received at least one prior treatment; 12 patients were treatment-naïve. The ORR at 24 weeks was 63.3% (95% CI, 43.9–80.1). The ORR by independent radiographic review using RECIST 1.1 was 66.7% (95% CI, 47.2–82.7). There did not appear to be any impact of PD-L1 status on the outcome; 58% of the PD-L1-positive and 71% of the PD-L1-negative patients had a response.

The most common treatment-related adverse events included diarrhea (83%), fatigue (70%), hypothyroidism (67%), stomatitis (60%), hypertension (57%), and nausea (57%). No new safety signals were observed. Treatment-related adverse events required a dose reduction for lenvatinib in 18 patients.

A phase 3 multicenter, open-label study (49) comparing lenvatinib plus pembrolizumab or lenvatinib plus everolimus vs. sunitinib in treatment-naive patients with aRCC is underway (NCT 02811861).

### Nivolumab Plus Cabozantinib

Cabozantinib is a multi-kinase TKI that inhibits VEGF receptors VEGFR1, VEGFR2, VEGFR3, AXL, RET, ROS1, TYRO3, MER, KIT, TRKB, FLT-3, and TIE-2. A phase 1 study to evaluate the tolerability and efficacy of nivolumab plus cabozantinib (NivoCabo) and nivolumab plus cabozantinib plus ipilimumab (NivoCabo+Ipi) in patients with metastatic urothelial carcinoma and other genitourinary malignancies was reported by Nadal et al. (50). Of the 75 patients enrolled, 7/47 patients treated with NivoCabo and 6/28 who recieved NivoCabo+Ipi had advanced renal cell carcinoma. Partial response was elicited in 7 patients with aRCC (50).

CheckMate9ER a phase 3 study (51) assessing the combination of nivolumab plus cabozantinib vs. suntinib in treatment-naive patients with aRCC is underway. Enrollment began in August 2017, and is ongoing (NCT 03141177).

## Discussion

The approval of combination immunotherapy with nivolumab plus ipilimumab marks the beginning of a new era in the therapeutic landscape for patients with advanced renal cell carcinoma. In addition, a wave of regulatory approvals with multiple VEGF inhibitor plus ICI combination is expected. While the preliminary data from these newer combinations are encouraging and hold great potential, they will also require new questions and concerns to be addressed as the existing treatment paradigm evolves.

The question of the choice and dose of VEGF inhibitor to use in combination with ICIs is important. The VEGF inhibitor should ideally support immunotherapy with positive immune modulation of the tumor micro-environment and be tolerable. While pazopanib was shown to have adequate VEGF inhibition and immunomodulatory activity, the tolerability has been marginal in combination with ICIs. This was initially observed in the CheckMate 016 phase 1 study that assessed the safety of nivolumab with TKIs, sunitinib or pazopanib or ipilimumab. Early hepatotoxicity noted in the dose-escalation phase of the nivolumab plus pazopanib arm required for the expansion phase to be aborted with this combination. Similar observation with pazopanib was made in the KEYNOTE-018, a phase 1/2 study that assessed the safety and efficacy of pazopanib and pembrolizumab in patients with aRCC (NCT02014636). Dose-limiting liver toxicity with grade 3–4 events were observed in 80–90% of the patients. It was determined that this combination was not feasible for further testing (52). In the Checkmate 016 study, greater efficacy with both ORR and durability of response was observed with the combination of nivolumab plus sunitinib, yet significant grade 3–4 toxicity precluded further development of the combination at the standard approved dose of sunitinib. Anti-VEGF therapy results in normalization of the vasculature that can reduce suppression in the tumor micro-environment (53). Using higher doses of anti-angiogenic agents may in fact result in hypoxia and decreased pH in the tumor micro-environment that are not conducive for optimal immune activity (54). The dose of VEGF inhibitors to achieve optimal modulation of the tumor micro-environment will need to be tailored (55). Therefore, future combination studies with VEGF inhibitors plus ICIs will need to address the question of optimizing the dose, ideally based on assessment of the TIME.

Increased incidence of higher-grade adverse events has been observed with the combinations of ICIs and VEGF inhibitors compared to monotherapy with the same agents. Toxicities attributed to VEGF inhibitors as well as ICIs have been described previously (56). Given the different underlying mechanisms causing these adverse events, immune-mediated for ICIs vs. direct drug-related for TKIs, the management strategies are very different and warrant clear understanding and education for both the patients and treating physicians. For optimal management appreciation of the above and accurate recognition of which component, ICI, or VEGF inhibitor is causative for a specific toxicity event will become critical, e.g., diarrhea may be the presenting symptom for ICI-induced auto-immune colitis but could also be drug-related to TKI therapy. Urgent treatment with steroids may be required for an auto-immune breakthrough toxicity; alternatively, the drug may simply need to be held for a few days for TKI-related symptoms. While not as critical as it was for high-dose IL-2, patient selection will require greater thought with combination therapy compared to monotherapy.

Interpretation of treatment response is another area of concern that may need reevaluation with newer combination therapy. Conventionally the RECIST criteria have been used for evaluation of response to chemotherapy and targeted therapy; iRECIST criteria were developed for evalution of response to immunotherapy. With the combination of two very different therapeutic modalities, new patterns of response or clinical benefit may emerge. Thought will need to be given to developing criteria that will capture the outcomes appropriately. We suggest this primarily because iRECIST criteria were developed only after new patterns of response were observed, as experience with ICIs accumulated. These considerations will become important for both interpretation of clinical trial data and application to clinical practice.

Another question that will need deliberation is how we should choose between dual-immune checkpoint inhibition of nivolumab/ipilimumab (for intermediate/poor-risk patients), the next wave of VEGF/ICI inhibitors, or sequential monotherapy with ICIs and VEGF inhibitors? Using the inevitable cross-trial comparison, ICI plus VEGF inhibitor combinations have elicited higher response rates (50%) and have a pronounced prolongation in PFS over sunitinib monotherapy, which makes the regulatory approval for many of these combinations very likely. However, none of these phase 3 VEGF/ICI combination trials incorporate a sequential TKI followed by PD-1 monotherapy comparison arm; thus, making it difficult to ascertain the true impact of moving the combination therapy upfront. In fact, only 25–30% of patients on the sunitinib arm of the recent phase 3 trials (Checkmate 214, IMmotion 151, Javelin 101) have had exposure to subsequent PD1 monotherapy. With several combinations to choose from, at present our leaning would be toward favoring dual-immune checkpoint therapy that has demonstrated a survival benefit. It will be important to follow the OS signal as the access to subsequent PD-1/PD-L1 therapy on the TKI control arms of the combination studies matures. At present there is paucity of prospective data as to how ICI-based immunotherapy and VEGF inhibition should be sequenced. Preliminary data from studies have shown activity for axitinib, sunitinib, and cabozantinib following treatment with ICIs (57–59). The notion of priming the tumor micro-environment with VEGF-targeted therapy followed by immunotherapy is intriguing as well (60). In other words, while the high response rates observed with VEGF/ICI combination are promising, it is unclear if we can interpret them as proof of true immunological synergy at this time. More data regarding the durability of responses and the impact on overall survival are critical to establish whether dual-immune checkpoint therapy or ICI plus VEGF inhibitor combinations with higher rate of adverse events, compared to optimally sequenced monotherapy, will become the preferred frontline standard.

Based on the different mechanisms of action for ICIs and TKIs, the choice of first-line therapy and the interpretation of outcomes after discontinuation of treatment due to toxicity will need to be put in appropriate perspective. Discontinuation of therapy secondary to intolerable toxicity with chemotherapy or targeted therapy may not be comparable to discontinuation of immunotherapy for high-grade auto-immune toxicity. Patients who receive ICIs and had their treatment discontinued for immune-mediated adverse events continue to maintain their response without requiring additional treatment, which is generally not the case for targeted therapy. This observation will need to be factored into decision-making for patients who respond to immunotherapy alone and may be spared VEGF pathway inhibition in the first line. These patients can potentially enjoy a significant treatment-free interval after they complete their treatment course or discontinue immunotherapy because of adverse events—some of these patients may never require further therapy. Analysis from the CheckMate 214 study reported by McDermott et al. showed the treatment-free survival after discontinuing nivolumab plus ipilimumab compared to sunitinib was different (42). The quality of response reported by Rini et al. from the same study confirmed that patients who received nivolumab plus ipilimumab continued to maintain responses if their treatment was discontinued for reasons other than progression (61). Additionally, the optimal duration of immunotherapy for patients who respond to immunotherapy and do not experience adverse events will need to be ascertained. Our current paradigm of continuing treatment until intolerable toxicity or progression of disease is derived from the chemotherapy experience. With immunotherapy-based treatment, the theoretical concerns of inducing resistance by immune-editing, antigenic drift, and irreversible T cell exhaustion after continuous exposure to immune modulation will need to be worked out.

There will certainly be clinical scenarios where eliciting high-response rates becomes critical. Symptomatic patients with high tumor burden, pending visceral crisis, or organ compromise are all scenarios where reliable and rapid cytoreduction, irrespective of mechanism (e.g., driven by VEGF, ICI, or a combined effect), is desirable and should be treated with VEGF/ICI combinations that reach into the 50–70% ORR range. On the other hand, many patients could just be exposed to combined ICI, which not only provides useful information on the responsiveness of different lesions but may also allow for excision and radiation of escape lesions or other future adaptive treatment strategies. This information is obviously lost in patients exposed to concurrent VEGF inhibitors.

Our impression is that the first-line combination treatment of aRCC will dichotomize between nivolumab/ipilimumab on the one hand, and potent VEGF/PD1 inhibitors (e.g., axitinib/pembrolizumab) on the other. The preference of one regimen vs. the other will likely depend on a multitude of factors including the country/health care system, clinical practice setting (academic vs. private practice), familiarity and experience, education, staffing, and patient's choice. Furthermore, most health care systems will have to ask the question of whether they should reimburse all regulatorily approved combinations or just focus on the one or two most promising regimens and try to save cost. This is certainly a question beyond the scope of this review but will undoubtedly have impact moving forward.

Another question in the changing landscape as several combinations are approved will be reaching consensus regarding the appropriate control arms for future studies? Which of these agents/combinations would serve as the most appropriate control? In this context, the optimal sequence for best therapeutic efficacy will need to be ascertained to ensure that the control arm does not compromise care. Over time the long-term follow-up data from dual ICI, VEGF/ICI, and sequential studies will help discern this from patient outcomes.

A multitude of options with potential to become therapeutic reality for patients with aRCC are moving steadily toward fruition. Exciting as this potential is, the new landscape poses new challenges, concerns, and questions that will need to be answered in a rational, thoughtful manner to best move the field forward. Ideally, biomarkers to predict response could help make the most optimal therapeutic choice, but despite intense efforts none have yet been identified. Expression of PD-L1 in the setting of aRCC has displayed mixed data and is not ready for use in clinical decision-making. Several approaches including evaluation of ctDNA and microbiome are under investigation.

## Author Contributions

All authors listed have made a substantial, direct and intellectual contribution to the work, and approved it for publication.

### Conflict of Interest Statement

AA has received honorarium from the following companies for speaking, or advisory boards: Bristol-Myers Squibb Company, Dynavax Technologies Corporation, Exelixis, Inc., Merck & Co., Inc., Novartis Pharmaceuticals Corporation, and Pfizer, Inc. and also reports that the following of the above companies fund clinical trial research that he is involved in: Bristol-Myers Squibb Company, Dynavax Technologies Corporation, Merck & Co., Inc. HH has received honorarium from the following companies for advisory roles: Armo Biosciences, Bristol-Myers Squibb Company, Merck & Co., Inc., Novartis Pharmaceuticals Corporation, Pfizer, Inc. and also reports that the following companies fund clinical trial research that he is involved in: Merck & Co., Inc., SFJ Pharmaceuticals. Furthermore, he receives clinical trail grant funding from Bristol-Myers Squibb Company.
